# Evaluation of Pancreatic Endocrine Reprogramming in Diabetic Cats

**DOI:** 10.3390/vetsci12121167

**Published:** 2025-12-08

**Authors:** Lune D. Geurts, Alice Zanon, Eylem E. Akyurek, Silvia Ferro, Roberta Sacchetto, Mila Della Barbera, Carolina Callegari, Gabriele Gerardi, Federico Fracassi, Hans S. Kooistra, Thomas A. Lutz, Eric Zini

**Affiliations:** 1 AniCura Dierenkliniek Rijngeest, President Kennedylaan 260, 2343 GX Oegstgeest, The Netherlands; lune.geurts@anicura.nl; 2 Department of Animal Medicine, Production and Health, University of Padova, viale dell’Università 16, 35020 Legnaro, Italy; alice.zanon@unipd.it (A.Z.); gabriele.gerardi@unipd.it (G.G.); 3 Department of Comparative Biomedicine and Food Sciences, University of Padova, viale dell’Università 16, 35020 Legnaro, Italy; eylememek.akyurek@unipd.it (E.E.A.); silvia.ferro@unipd.it (S.F.); roberta.sacchetto@unipd.it (R.S.); 4 Department of Cardiac, Thoracic and Vascular Science, Cardiovascular Pathology Section, University of Padova, via A. Gabelli 61, 35121 Padova, Italy; mila.dellabarbera@unipd.it; 5 IDEXX Laboratories Italia, via Guglielmo Silva 36, 20149 Milano, Italy; carolina-callegari@idexx.com; 6 Department of Veterinary Medical Science, University of Bologna, via Tolara di Sopra 43, 40064 Ozzano dell’Emilia, Italy; federico.fracassi@unibo.it; 7 Department of Clinical Sciences, Faculty of Veterinary Medicine, Utrecht University, Yalelaan 108, 3584 CM Utrecht, The Netherlands; h.s.kooistra@uu.nl; 8 Institute of Veterinary Physiology, Vetsuisse Faculty, University of Zurich, Winterthurerstrasse 260, 8057 Zurich, Switzerland; thomas.lutz@uzh.ch; 9 Clinic for Small Animal Internal Medicine, Vetsuisse Faculty, University of Zurich, Winterthurerstrasse 260, 8057 Zurich, Switzerland; 10 AniCura Istituto Veterinario Novara, Strada Provinciale 9, 28060 Granozzo con Monticello, Italy

**Keywords:** α-cell, β-cell, development, diabetes mellitus, feline, islets

## Abstract

Transcription factors control the development of the endocrine pancreas in various mammals. In humans, paired box-4 (PAX4) and aristaless-related homeobox (ARX) allocate endocrine progenitor cells toward β-cell and α-cell specification, respectively. In adulthood, PAX4 contributes to reprogramming α-cells into β-cells and exocrine into endocrine cells; induction of ARX in β-cells drives them to reprogram into α-cells. Feline diabetes mellitus has a similar pathophysiology to human type 2 diabetes, but information about the role of these transcription factors is unavailable in diabetic cats. The study aim was to test whether diabetic cats have an increased number of pancreatic cells expressing developmental markers of β- and α-cells, respectively, suggesting reprogramming. In 9 diabetic and 9 well-matched control cats, pancreas was collected, formalin-fixed and paraffin-embedded. Tissue slides were labelled for insulin, glucagon, PAX4, and ARX. Positive cells for each marker and double-positive cells for their combinations were counted in the pancreas and compared between groups. Against controls, diabetic cats had fewer insulin-positive cells in the islets (*p* = 0.001) and exocrine pancreas (*p* = 0.038); glucagon-positive cells were similar. In the islets, diabetic cats had higher counts of insulin/glucagon-positive cells (*p* = 0.024), PAX4-positive cells (*p* = 0.038), as well as PAX4/insulin-positive cells (*p* = 0.027). In conclusion, in diabetic cats, the increased number of islet cells expressing PAX4 leads to the hypothesis that β-cells change to an earlier stage of differentiation or that novel β-cells are formed. Furthermore, the higher count of islet insulin/glucagon-positive cells might indicate that α-cells transform into β-cells or vice versa. Hence, reprogramming seems possible in diabetic cats, specifically in the islets.

## Simple Summary

Transcription factors, including paired box-4 (PAX4) for β-cells and aristaless-related homeobox (ARX) for α-cells, control the development of the endocrine pancreas in humans. Diabetes mellitus in cats resembles type 2 diabetes in humans, but information about these transcription factors is unavailable. In 9 diabetic and 9 control cats, pancreatic sections were immunostained for insulin, glucagon, PAX4, and ARX. Positive cells for each marker were counted and compared. Diabetic cats had significantly fewer insulin-positive cells in the islets and exocrine pancreas compared with controls. In the islets, diabetic cats had significantly more insulin and glucagon double-positive cells, PAX4 positive cells, and PAX4 and insulin double-positive cells. In conclusion, in diabetic cats, the augmented number of islet cells expressing PAX4 might suggest that β-cells change to an earlier stage of differentiation or that new β-cells are formed. The increased number of islet insulin and glucagon double-positive cells indicates that α-cells are transforming into β-cells or vice versa.

## 1. Introduction

During embryogenesis in different mammals, the nuclear transcription factor paired box-4 (PAX4) allocates endocrine progenitor cells toward β-cells, whereas aristaless-related homeobox (ARX) allocates endocrine progenitor cells toward α-cells; a competitive reciprocal interaction between PAX4 and ARX is involved in the decision of cell fate between β-cells and α-cells [1,2,3,4,5]. In human adulthood and rodents, PAX4 is crucial for β-cell viability throughout life [2,3]. Indeed, mutations associated with decreased activity of the gene encoding PAX4 are linked to type 2 diabetes mellitus (DM), and overexpression of PAX4 in β-cells stimulates their proliferation in both humans and rats [3,4]. Furthermore, inducing the expression of PAX4 in α-cells of rodents was shown to initiate the reprogramming of α-cells into β-cells [5]. In healthy adult humans, ARX expression remains restricted to α-cells [6]. In contrast, in type 2 DM, ARX was also observed in β-cells, which reprogrammed from β-cells into α-cells [7]. Of note, the plasticity of islet cells conferred by PAX4 and ARX has become a major research focus in human diabetes research because their targeting may prompt the replenishment of β-cells [8].

In addition to islets, cells of the exocrine pancreas also seem to constitute important candidates for β-cell regeneration in humans because they can reprogram into insulin-producing cells, mostly in pancreatic ducts [9]. Culturing human acinar cells overexpressing PAX4 in combination with the suppression of ARX enhances the production of insulin-secreting β-like cells [10].

Although the pathophysiology of type 2 DM in humans and feline DM is rather similar, it is unknown whether reprogramming of endocrine and exocrine pancreatic cells occurs in diabetic cats. However, based on comparable findings in different species, these regulatory pathways are likely evolutionarily conserved across mammals and expected to also occur in cats. In a recent investigation, it was found that diabetic cats, in comparison to well-matched controls, had an increased number of cells located in the exocrine pancreas that were positive for proliferating cell nuclear antigen, a marker of proliferation and DNA repair, in nearby islets [11]. It was hypothesized that they represent acinar or progenitor cells with intense nuclear activity, possibly undergoing reprogramming into endocrine cells.

The aim of the study was to verify whether cells of the pancreatic islets and exocrine pancreas more frequently express developmental markers of α-cells and β-cells in diabetic cats compared with controls, suggesting reprogramming.

## 2. Materials and Methods

### 2.1. Cases and Controls

In total, 18 cats were included: 9 with DM, and 9 well-matched controls. Diabetic cats that met the following criteria were prospectively included in the study: (i) DM was diagnosed based on established clinical and laboratory findings, including increased serum fructosamine concentrations [12]; (ii) cats died spontaneously or were euthanized at the authors’ institutions between January 2016 and June 2017; (iii) a postmortem examination was carried out within 1 h after death; (iv) pancreas samples of approximately 1 cm in diameter were collected from the caudal tip of the left tail; and (v) informed consent was signed by all owners. Randomly selected cats with a similar distribution of age, sex, breed, and body weight that died spontaneously or were euthanized because of another disease during the same corresponding period at the same institutions and with pancreatic samples collected similarly to diabetic cases were used as controls. In these cats, DM was ruled out based on medical history, physical examination, and blood work, including normal serum fructosamine concentrations.

### 2.2. Tissue Preparation and Histopathology

Pancreatic samples were fixed in 10% neutral buffered formalin for 5 days and then embedded in paraffin. From each sample, 10 consecutive 3 to 4 µm thick sections were cut and mounted on microscope slides (Superfrost Plus, Menzel GmbH, Braunschweig, Germany). An automatic staining system (Leica Autostainer XL, Leica Biosystems, Milan, Italy) was used for hematoxylin and eosin. Sections were deparaffinized and rehydrated in descending alcohol solutions. After washing in distilled water, sections were stained in a filtered solution containing 25% Mayer hematoxylin and 75% Carazzi hematoxylin (Sigma-Aldrich, Milan, Italy) for 7 min, rinsed in running tap water for 5 min, stained with eosin for 1 min, dehydrated in ascending alcohol solutions, and cleared in xylene.

Bennhold’s Congo red (Sigma-Aldrich) was also used. Slides were deparaffinized and hydrated in distilled water. Then, sections were stained in Congo red solution for 30–60 min, rinsed in distilled water, differentiated rapidly (5–10 dips) in alkaline alcohol solution, rinsed in running tap water for 5 min, counterstained in Gill’s hematoxylin (Sigma-Aldrich) for 30 s, and rinsed in tap water for 1 min. Thereafter, sections were dipped in ammonia water for 30 s, rinsed in tap water for 5 min, dehydrated through 95% alcohol solution, and cleared in xylene.

### 2.3. Immunofluorescence

Double-labelling immunofluorescence was carried out using standard methods [13] on formalin-fixed, paraffin-embedded pancreas for the following combinations of primary antibodies: insulin (polyclonal rabbit anti-insulin, #4590, Cell Signaling Technology, Danvers, MA, USA) and glucagon (polyclonal rabbit anti-glucagon, #2760, Cell Signaling Technology), insulin and PAX4 (polyclonal rabbit anti-PAX4, PA5-13649, Thermo Fisher Scientific Invitrogen, Waltham, MA, USA), insulin and ARX (monoclonal mouse anti-ARX, 11F6.2, Merck, Darmstadt, Germany), glucagon and PAX4, glucagon and ARX, and PAX4 and ARX. The dilution of the primary antibodies for insulin, glucagon, and ARX was 1:250, and for PAX4 was 1:50. Biotinylated goat anti-rabbit IgG antibody (BA-1000, Vector Laboratories, Burlingame, CA, USA) was used to amplify the signal of primary rabbit antibodies, and biotinylated goat anti-mouse IgG antibody (BA-9200, Vector Laboratories) was used for primary mouse antibodies. For their binding, an avidin/biotin peroxidase system was applied (PK-6100, Vectastain Elite ABC HRP Kit, Vector Laboratories). The slides were then incubated with a biotinylated tyramide solution to increase binding and achieve more signal amplification. Later, the secondary antibody Alexa Fluor 647 streptavidin conjugated (#016-600-084, Jackson Immuno Research Laboratories Inc. West Grove, PA, USA) was used for primary antibodies showing signal amplification, and the secondary anti-mouse antibody Alexa Fluor 555 donkey anti-rabbit (A-31572, Thermo Fisher Scientific Invitrogen, Milan, Italy) was used for primary antibodies that had no signal amplification. Normal goat serum and normal donkey serum were used as blocking agents, obtained by diluting 100 µL of serum in 10 mL 0.3% PBTX (Triton X-100 in phosphate-buffered saline), during application of primary and secondary antibodies, as well as during amplifications. For each slide, 500 µL of fitting solution was applied on glass incubation tables. Finally, a DAPI counterstain (Art. No. 6335.1, Carl Roth, Karlsruhe, Germany) was applied, incubating slides for 4 min with a 1:1000 solution in PBS.

A heat-mediated antigen retrieval technique was used for all reactions. The slides were incubated with a buffer in plastic Coplin staining jars for 20 min in a 95 °C antigen retrieval steamer. For reactions including anti-ARX antibody, a Tris-EDTA pH 9.0 buffer (10 mM Tris base, 1 mM EDTA solution, 0.05% Tween 20) was used. For those that did not include the anti-ARX antibody, a sodium citrate 0.05% Tween pH 6.0 antigen retrieval buffer (10 mM Sodium citrate, 0.05% Tween 20) was used. Because the antibodies for PAX4 (polyclonal rabbit) and ARX (monoclonal mouse) had not been previously used in cats, to obtain positive controls and assess specificity, available pancreatic tissue of healthy rabbits and mice was used.

The same microscope used above for hematoxylin and eosin sections was used with the following 3 channels: to detect the secondary antibody Alexa Fluor 647, the Zeiss filter set 43 was used with excitation BP 545/25, beam splitter FT 570, and emission BP 605/70; for the secondary antibody Alexa Fluor 555, the Zeiss filter set 50 with excitation BP 640/30, beam splitter FT 660, and emission BP 690/50 was used; and for DAPI counterstain, the Zeiss filter set 49 was used with excitation G 365, beam splitter FT 395, and emission BP 445/50.

For analysis, from every slide of each reaction, pictures at magnification 400× were collected using the above 3 microscopy channels. Specifically, 10 different pictures were taken from fields containing 1 pancreatic islet and 10 from fields without pancreatic islets, randomly moving the microscope over the slide. Islets with a major axis smaller than 5 endocrine cells were arbitrarily excluded. Pictures with large vessels, ducts, and interlobular tissue were also excluded from measurements. Analyses were performed by 2 pathologists, and any discrepancy was resolved by consensus.

### 2.4. Islet Morphology and Cell Counts

Sections stained with hematoxylin and eosin were evaluated to identify morphologic lesions of the pancreatic islets, and those with Congo red to examine pancreatic amyloid in cats with and without DM. If present, the amount of amyloid was scored as mild if the cross-sectional area of the islet with deposits was below one-third and as moderate-to-severe if above. In addition, nodular hyperplasia was assessed in both groups, and if present, the corresponding picture was not considered for analysis of the endocrine pancreas. Regarding immunohistochemistry, ImageJ2 software (http://rsb.info.nih.gov/ij/ accessed on 30 April 2021) was used to quantify positively-stained cells in all cats. The counting method was based on the manual cell counter plugin of the software. The number of cells expressing insulin, glucagon, ARX, PAX4, or any of their combinations was counted in each picture, and the average was calculated for both islets and the exocrine pancreas.

### 2.5. Statistical Analysis

The results are presented as median and range. To check the process of matching diabetic and control cats, the age, sex, breed, and body weight of cats in the 2 groups were compared using Fisher’s exact test and the Mann–Whitney U test. Differences between diabetic and control cats were investigated for cells single-labelled for insulin, glucagon, ARX, and PAX4 in the islets and exocrine pancreas using the Mann–Whitney U test. Differences were also explored for the number of double-labelled cells for insulin and glucagon, ARX and insulin, ARX and glucagon, ARX and PAX4, PAX4 and insulin, and PAX4 and glucagon in the islets and exocrine pancreas, as above. Additionally, the ratio between cells double-labelled for PAX4 and insulin and cells single-labelled for insulin was calculated and compared between groups with the same test. The proportion of cats with islet amyloid deposits and with nodular hyperplasia was compared with Fisher’s exact test. Significance was considered at *p* < 0.05. The analyses were conducted with commercial software (SPSS version 24.0, IBM, Armonk, NY, USA).

## 3. Results

### 3.1. Cases and Controls

The 9 diabetic cats consisted of 7 castrated males and 2 neutered females, with a median age of 11 years (range: 7–16) and a median body weight of 4.5 kg (range: 3.3–5.8). Seven were domestic shorthair cats and 2 purebred, including one Maine Coon and one Siamese. Concurrent diseases diagnosed along with DM were hypersomatotropism in 2 cats and hyperadrenocorticism, pancreatitis, renal failure, cholangitis, and meningioma in 1 cat each. Ketoacidosis was identified in 2 cats. Median serum fructosamine concentration, recorded within 2 weeks before death, was 619 µmol/L (range: 556–704; normal: <340). Among the 9 control cats, there were 5 castrated males and 4 females, including 3 neutered, with a median age of 11.8 years (range: 8–17) and a median body weight of 4.1 kg (range: 2.2–6.1). All were domestic shorthair cats. Diseases recorded at death were idiopathic chylothorax, idiopathic myositis, liver carcinoma, lung carcinoma, lung strongylosis, multicentric lymphoma, nasal carcinoma, right arrhythmogenic cardiomyopathy, and seizure caused by suspected intoxication. Median serum fructosamine concentration was 297 µmol/L (range: 272–321). Population characteristics did not differ between the 2 groups.

### 3.2. Islet Morphology

**Figure 1:**
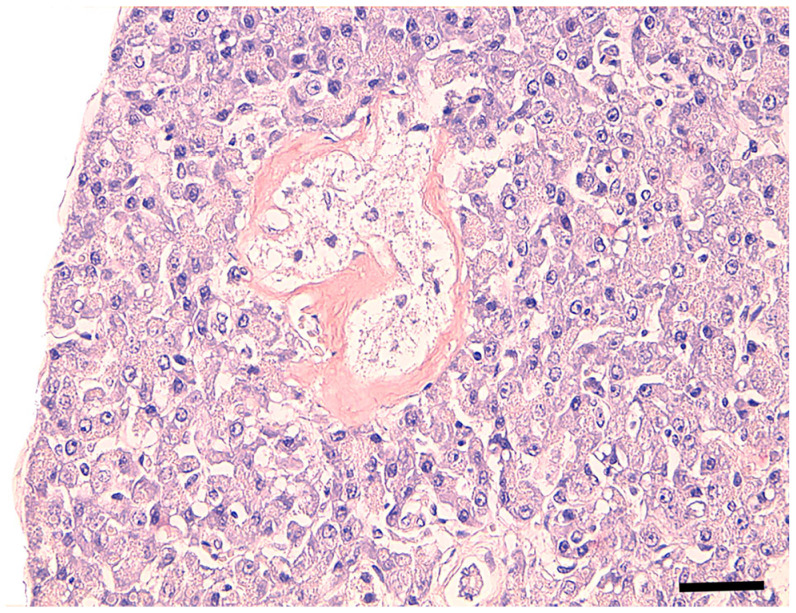
Pancreatic islet, cat. Diabetic cat with hydropic degeneration of islet cells and Congo-red-positive amorphous material (amyloid) deposits in the islet periphery. Scale bar = 40 µm. Congo red stain.

Examination of pancreatic sections showed differences in the morphology of the islet cells between diabetic and control cats. Islet cells with clear cytoplasm, compatible with hydropic degeneration (Figure 1), were observed in 3 of the diabetic cats but in none of the control cats.

The nuclei of the islet cells appeared morphologically similar in both groups based on subjective qualitative assessment. Amyloid deposits were observed in pancreatic islets of 4 diabetic cats and of 3 control cats (*p* = 1.000) and were mild in each case. Pancreatic nodular hyperplasia of the acinar cells of the exocrine pancreas was observed in 2 diabetic cats and in 2 control cats (*p* = 1.000). Nodules were scattered in the exocrine pancreas and did not contain endocrine tissue. Morphologic description of islets and exocrine pancreas of diabetic cats is provided in Table 1.

### 3.3. Cell Counts

As expected, diabetic cats had fewer insulin-positive cells per islet compared with controls (median: 14.8, range: 2.4–51, vs. median: 64, range: 33.4–71.4, *p* = 0.001); the number of islets was similar between groups. Diabetic cats also had fewer insulin-positive cells in aggregates scattered in the exocrine tissue compared with control cats (median: 5, range: 1.8–14.6, vs. median: 10, range: 4.4–19, *p* = 0.038).

The number of glucagon-positive cells did not differ between cats with and without DM, neither in the islets nor in the exocrine pancreas.

**Figure 2:**
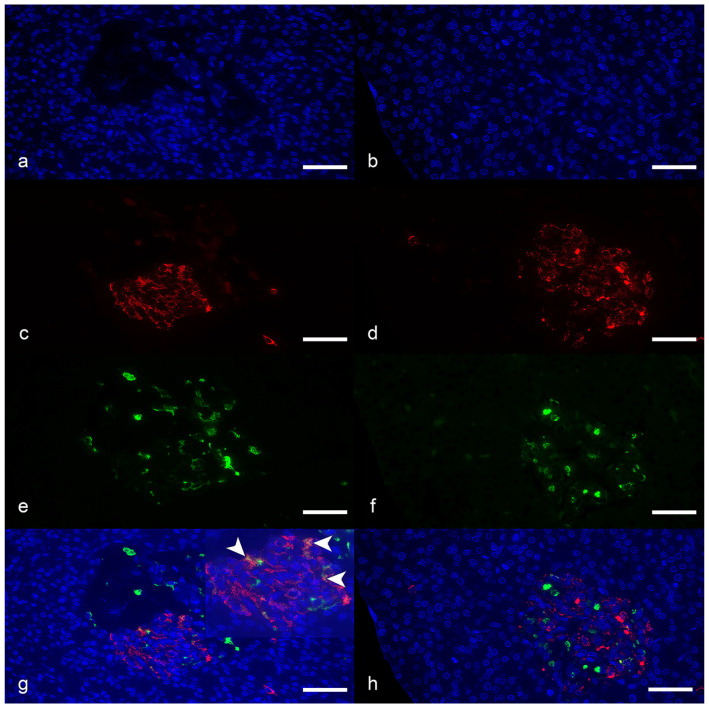
Representative immunofluorescence images of pancreatic islets from diabetic (**a**,**c**,**e**,**g**) and control cats (**b**,**d**,**f**,**h**); scale bars = 30 µm. Double-labelling immunofluorescence for insulin ((**b**,**f**), red fluorescence) and glucagon ((**c**,**g**), green fluorescence). Nuclei were revealed by DAPI (**a**,**b**). Merged images showing co-localization of the fluorochromes are displayed in (**d**,**h**). Arrowheads indicate cells co-expressing insulin and glucagon (yellow) in the islet from a diabetic cat (**d**).

The total number of cells double-labelled for insulin and glucagon was low, but diabetic cats had a higher count per islet compared with controls (median: 0.2, range: 0–11, vs. median: 0, range: 0–0.4, *p* = 0.024) (Figure 2 and Figure 4a).

Islet cells double-labelled for insulin and glucagon were observed in 8 of 9 diabetic cats and in 3 of 9 controls (Fisher’s exact test, *p* = 0.049). Their count was not different in the exocrine pancreas.

The number of PAX4-positive cells per islet was higher in the diabetic group than in controls (median: 9.6, range: 0–29.6, vs. median: 2.2, range: 0.2–6.4, *p* = 0.038), whereas in the exocrine pancreas it did not differ.

**Figure 3:**
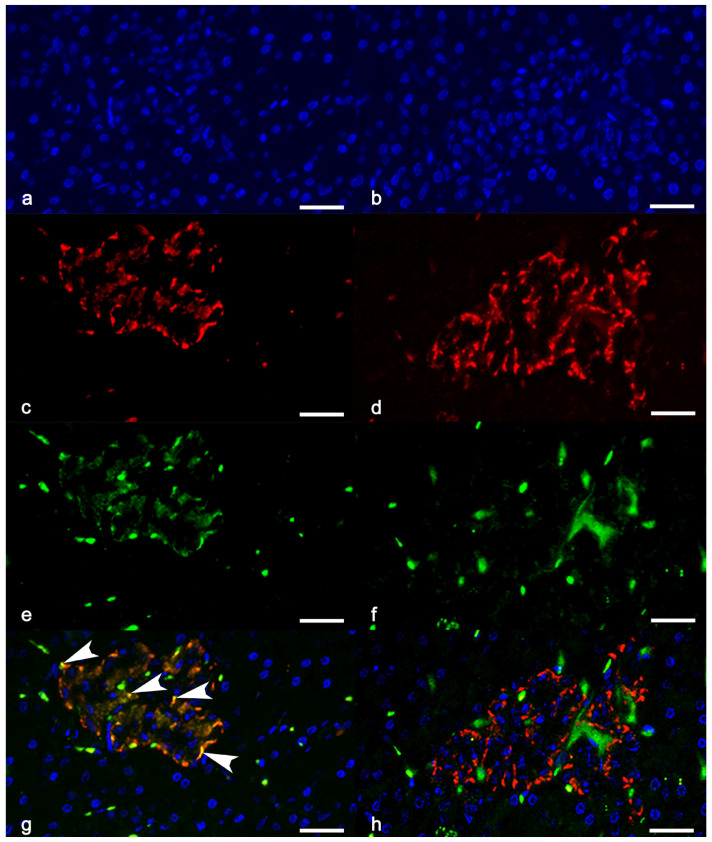
Representative immunofluorescence images of pancreatic islets from diabetic (**a**,**c**,**e**,**g**) and control cats (**b**,**d**,**f**,**h**), scale bars = 25 µm. Double-labelling immunofluorescence for insulin ((**b**,**f**), red fluorescence) and nuclear transcription factor PAX4 ((**c**,**g**), green fluorescence). Nuclei were revealed by DAPI (**a**,**e**). Merged images showing co-localization of the fluorochromes are displayed in d and h. Arrowheads indicate cells co-expressing PAX4 and insulin (yellow) in the islet from a diabetic cat (**d**).

**Figure 4:**
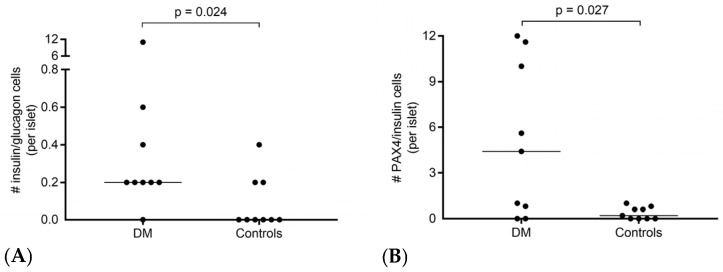
Dot plots. (**A**) An increased count of cells double-labelled for insulin and glucagon in the islets of 9 diabetic cats compared with 9 control cats. (**B**) An increased count of cells double-labelled for PAX4 and insulin in the islets of 9 diabetic cats compared with 9 control cats.

Regarding the cells double-labelled for PAX4 and insulin, diabetic cats had a higher number per islet (median: 4.4, range: 0–12, vs. median: 0.2, range: 0–1, *p* = 0.027) (Figure 3 and Figure 4B), whereas differences were not significant in the exocrine pancreas.

The ratio between islet cells double-labelled for PAX4 and insulin and those single-labelled for insulin was also higher in diabetic cats compared with controls (median: 0.2, range: 0–4.2, vs. median: 0, range: 0–0, *p* = 0.015).

The number of cells double-labelled for PAX4 and glucagon did not differ, both in the islets and in the exocrine pancreas. The count of ARX-positive cells did not differ between diabetic and control cats in both pancreatic components. In addition, no significant difference was found regarding cells double-labelled for ARX and insulin, ARX and glucagon, as well as ARX and PAX4 in the islets and in the exocrine pancreas. Cell counts for each reaction are provided in Table 2.

## 4. Discussion

The observation of a 75% reduction in the number of insulin-positive cells in islets and a 50% cell reduction in aggregates scattered in the exocrine pancreas of the diabetic cats is similar to the results of a previous investigation showing 65% lower counts in cats with DM [13]. This was to be expected based on the fact that β-cell loss is an important component of the pathophysiology of the disease [14,15]. Similar to the previous study [14], the number of glucagon-positive cells did not differ between diabetic and control cats. In contrast, O’Brien and co-workers found a reduction in α-cells in cats with DM compared with controls [15]. The potential sampling variability, exclusion of large islets, and heterogeneity in islet distribution across the pancreas might have played a role in accounting for this discrepancy. Of note, in humans with type 2 DM, hyperplasia of α-cells is common and seems to play a protective role by promoting β-cell proliferation [8].

The count of cells double-labelled for insulin and glucagon in the islets was increased in diabetic cats. This result might suggest a bidirectional transformation between the α-cells and β-cells of the pancreas. In humans with type 2 DM, different islet cells, including α-cells, are able to differentiate into mature β-cells, and insulin-producing cells can also reprogram into glucagon-producing cells [2,7]. Hence, the phenotype of the α-cells and β-cells can become flexible in the diabetic milieu, in cats as well. Of note, 1 of the diabetic cats had a remarkably high number of cells double-labelled for insulin and glucagon compared with the other affected cases. In addition to diabetes, the cat had acromegaly. In the other diabetic cat with acromegaly in this series, the count of cells double-labelled for insulin and glucagon was not particularly high, and thus the reason for the above finding remains unknown. Alternatively, double-labelled for insulin and glucagon, such cells may also arise from metabolic stress [16], immature endocrine phenotypes [17], or, although less likely, technical artefacts associated with double immunofluorescence.

Cats with DM had a higher count of PAX4-positive cells in the islets. PAX4 is a transcription factor of the early differentiation phase of the β-cell lineage; in particular, it is a critical regulator of embryonic development of β-cells and supports their plasticity in adult islets [18]. Additionally, diabetic cats had more islet cells concurrently labelled for PAX4 and insulin, despite a reduction in the overall number of β-cells, possibly indicating that cells expressing PAX4 and those expressing PAX4 and insulin represent 2 different stages of β-cell differentiation, thus pointing to reprogramming. Alternatively, these results may indicate that newly formed β-cells arise from progenitors located within the islets of diabetic cats. Of note, cells that were concurrently labelled for PAX4 and insulin were also observed in control cats. Whether or not this is associated with the normal turnover of β-cells is unknown.

No differences were found concerning the number of ARX-positive cells. ARX is a transcription factor that marks the α-cell lineage. Its absence leads to a reduction in α-cells and an increased number of β- and δ-cells [19]. This suggests that ARX promotes the generation of α-cells, antagonizing β- and δ-cells during embryogenesis [1]. In diabetic humans, an insulin-positive cell that displays progenitor markers of α-cells (e.g., ARX) but not glucagon may represent a β-cell undergoing reprogramming into an α-cell [7].

No substantial group differences were found for cells double-labelled for ARX and insulin and for PAX4 and glucagon in either islets or the exocrine pancreas. This is potentially in contrast to our findings regarding increased islet cells concurrently expressing insulin and glucagon. Thus, even if the 2 cell lineages can partly merge in diabetic cats, this does not seem to involve PAX4 or ARX, and other unexplored markers and pathways might be implicated.

There are some limitations to this study that need to be addressed. Overall, the sample size was relatively small, possibly reducing the chance of identifying differences between groups. In addition, the control group included cats with different diseases; however, including healthy matched cases was not possible for ethical reasons. It cannot be excluded that some of these disorders had resulted in insulin resistance [20], which may have affected pancreatic histology and endocrine cell phenotypes. In particular, the markedly increased double-labelled cell count in the cat with DM and acromegaly raises the possibility of disease-specific effects.

## 5. Conclusions

In diabetic cats, the increased number of islet cells expressing PAX4 leads to the hypothesis that β-cells may change to an earlier stage of differentiation or that new β-cells are formed. Furthermore, the increased number of islet cells concurrently expressing insulin and glucagon might indicate that α-cells are transforming into β-cells or vice versa. Therefore, reprogramming seems possible in diabetic cats, specifically in the islets.

## Figures and Tables

**Table 1:** Cats with DM, histopathology of the pancreas.

Cat	Microscopic Findings
1	Pancreatic islet cells with hydropic degeneration and amyloid deposits in the islets; exocrine pancreas with normal morphology.
2	Pancreatic islet cells with normal morphology; nodular hyperplasia scattered in the exocrine pancreas.
3	Pancreatic islet cells with normal morphology, amyloid deposits in the islets; exocrine pancreas with normal morphology.
4	Pancreatic islet cells with hydropic degeneration; exocrine pancreas with normal morphology.
5	Pancreatic islets and exocrine pancreas with normal morphology.
6	Pancreatic islet cells with normal morphology, amyloid deposits in the islets; nodular hyperplasia scattered in the exocrine pancreas.
7	Pancreatic islets and exocrine pancreas with normal morphology.
8	Pancreatic islets with amyloid deposits; exocrine pancreas with normal morphology.
9	Pancreatic islet cells with hydropic degeneration; exocrine pancreas with normal morphology.

**Table 2:** Number of immunolabelled cells in the islets and exocrine pancreas of diabetic and control cats.

Cell Type	DM Median (Range)	Controls Median (Range)	*p*-Value
***Pancreatic islets:***			
Insulin	14.8 (2.4–51)	64 (33.4–71.4)	*p* = 0.001
Glucagon	9.9 (0.9–14.4)	9.5 (5.6–14.3)	*p* = 0.965
Insulin and glucagon	0.2 (0–11)	0 (0–0.4)	*p* = 0.024
PAX4	9.6 (0–29.6)	2.2 (0.2–6.4)	*p* = 0.038
PAX4 and insulin	4.4 (0–12)	0.2 (0–1)	*p* = 0.027
PAX4 and glucagon	3.6 (0–17)	0.8 (0–2.8)	*p* = 0.084
ARX	2.6 (0.8–4.6)	2.8 (1.2–13.2)	*p* = 0.657
ARX and insulin	0 (0–0.4)	0 (0–0.2)	*p* = 0.634
ARX and glucagon	0 (0–1.4)	0.2 (0–1.2)	*p* = 0.307
ARX and PAX4	0 (0–0.2)	0 (0–0.2)	*p* = 0.999
***Exocrine pancreas:***			
Insulin	5 (1.8–14.6)	10 (4.4–19)	*p* = 0.038
Glucagon	0.5 (0.1–1.4)	1 (0.3–1.6)	*p* = 0.352
Insulin and glucagon	0.8 (0–2)	0 (0–1.2)	*p* = 0.138
PAX4	2.2 (1–4.4)	1 (0–3)	*p* = 0.101
PAX4 and insulin	0.4 (0–3.4)	0.2 (0–1.2)	*p* = 0.298
PAX4 and glucagon	1.2 (0.2–1.6)	0.4 (0–2.2)	*p* = 0.099
ARX	21.6 (4.8–40.4)	11.8 (2.2–26)	*p* = 0.112
ARX and insulin	0 (0–0.4)	0 (0–0.2)	*p* = 0.634
ARX and glucagon	0 (0–0)	0 (0–0.8)	*p* = 0.029
ARX and PAX4	0 (0–1)	0 (0–0.2)	*p* = 0.634

## Data Availability

The original contributions presented in this study are included in the article. Further inquiries can be directed to the corresponding author.

## Abbreviations

The following abbreviations are used in this manuscript:

DM	Diabetes mellitus
PAX4	Paired box-4
ARX	Aristaless-related homeobox
